# A qualitative study exploring healthcare workers’ lived experiences of the impacts of COVID-19 policies and guidelines on maternal and reproductive healthcare services in the United Kingdom

**DOI:** 10.18332/ejm/171802

**Published:** 2023-11-08

**Authors:** Jonathan Chaloner, Irtiza Qureshi, Mayuri Gogoi, Winifred C. Ekezie, Amani Al-Oraibi, Fatimah Wobi, Joy O. Agbonmwandolor, Laura B. Nellums, Manish Pareek

**Affiliations:** 1 Department of Respiratory Sciences, University of Leicester, Leicester, United Kingdom; 2 Lifespan and Population Health, School of Medicine, University of Nottingham, Nottingham, United Kingdom; 3 Diabetes Research Centre, University of Leicester, Leicester, United Kingdom; 4 Department of Sociology and Policy, School of Social Sciences and Humanities, Aston University, Birmingham, United Kingdom; 5 Public Health Institute, Liverpool John Moores University, Liverpool, United Kingdom; 6 Nottingham University Hospitals NHS Trust, Nottingham, United Kingdom; 7 College of Population Health, University of New Mexico, Albuquerque, Mexico; 8 Department of Infection and HIV Medicine, University Hospitals of Leicester NHS Trust, Leicester, United Kingdom; 9 NIHR Leicester Biomedical Research Centre, Leicester, United Kingdom

**Keywords:** healthcare workers, vaccine hesitancy, COVID-19, policies and guidelines, redeployment, infection controls

## Abstract

**INTRODUCTION:**

During the COVID-19 pandemic, pregnant women were regarded as vulnerable to poor health outcomes if infected with the SARS-CoV-2 (COVID-19) virus. To protect the United Kingdom’s (UK) National Health Service (NHS) and pregnant patients, strict infection control policies and regulations were implemented. This study aimed to understand the impact of the COVID-19 policies and guidelines on maternal and reproductive health services during the pandemic from the experiences of healthcare workers (HCWs) caring for these patients.

**METHODS:**

This qualitative study involved HCWs from the United Kingdom Research study into Ethnicity and COVID-19 outcomes in Healthcare workers (UK-REACH) project. Semi-structured interviews and focus groups were conducted online or by telephone with 44 diverse HCWs. Transcripts were thematically analyzed following Braun and Clarke’s principles of qualitative analysis.

**RESULTS:**

Three key themes were identified during analysis. First, infection control policies impacted appointment availability, resulting in many cancellations and delays to treatment. Telemedicine was also used extensively to reduce risks from face-to-face consultations, disadvantaging patients from minoritized ethnicities. Secondly, staff shortages and redeployments reduced availability of consultations, appointments, and sonography scans. Finally, staff and patients reported challenges accessing timely, reliable and accurate information and guidance.

**CONCLUSIONS:**

COVID-19 demonstrated how a global health crisis can impact maternal and reproductive health services, leading to reduced service quality and surgical delays due to staff redeployment policies. Our findings underscore the implications of policy and future health crises preparedness. This includes tailored infection control policies, addressing elective surgery backlogs early and improved dissemination of relevant vaccine information.

## INTRODUCTION

On 5 March 2020, the United Kingdom (UK) reported its first death of a coronavirus disease 2019 (COVID-19) patient in a Berkshire hospital^1^, triggering a cascade of government policies and guidelines aimed to safely protect ‘vulnerable’^2^ patients at risk of exposure to COVID-19. This policy was prioritized to ‘protect the NHS (National Health Service)’^3^ from overburdening and overstretching of the service to respond effectively to the pandemic.

Concerns initially arose regarding the risks of COVID-19 to pregnant women, their unborn babies and neonates. Comparisons were drawn between the risks of COVID-19 and the outcomes in pregnancy with previous respiratory infections, including the Middle Eastern Respiratory Syndrome Coronavirus (MERS-CoV) and Severe Acute Respiratory Syndrome Coronavirus 1 (SARS-CoV-1)^4-6^. The potential vertical transmission of COVID-19 from mother-to-fetus was unknown^7^. Emerging evidence indicated that pregnant women from ethnic minority backgrounds faced increased vulnerability^8^ due to social and cultural determinants of health affecting these populations^9,10^.

The NHS implemented various infection control provisions to protect staff and patients. These provisions included the use of personal protective equipment (PPE), twice weekly lateral flow tests for healthcare workers (HCWs); enforcing social distancing; restrictions on the number of visitors allowed into hospitals; and minimizing face-to-face consultations and HCW exposure to patients^11^.

Although these policies and guidelines were uniformly introduced across the NHS, their impact varied in all treatment specialities. In maternal and reproductive health services (MRH), staff reported challenges providing women-centered care, handling increased workload, trust barriers providing care while wearing PPE, and uncertainty with rapidly changing guidelines^12-14^.

Visitor restrictions also made decision making more difficult for pregnant women, further adding to the ‘stress’, ‘trauma’ and ‘difficulty’ of pregnancy^15^. It was also reported that some people of minoritized ethnicities opted for private healthcare charges to enable their partners to be present during antenatal care (ANC) and during labor^15,16^. However, the impact of the UK Government’s COVID-19 policies and guidelines on maternal and reproductive healthcare staff and services largely remains understudied. To address this gap, we undertook a retrospective qualitative study using the the United Kingdom Research study into Ethnicity And COVID-19 outcomes in Healthcare workers (UK-REACH) fourth work package (WP4) dataset. Our aim was to understand how the COVID-19 policies and guidelines impacted MRH staff and services, and provide insights for midwifery services in future respiratory pandemics.

## METHODS

### Study design and setting

This qualitative study is a subset of the UK-REACH project, focusing on the impact of COVID-19 policies and guidelines on reproductive and maternal healthcare workers during the pandemic. The study’s methods, design, data collection and analysis have previously been detailed in the study protocol^17,18^.

### Study population

We collected the data between December 2020 and July 2021 from clinical and non-clinical HCWs (≥16 years old). The sample for the wider study was purposively recruited to ensure diversity and underserved populations. Participants were recruited into the study through NHS Trusts, the General Medical Council (GMC), General Optical Council (GOC), Health and Care Professions Council (HCPC), British Association of Physicians of Indian Origin (BAPIO), Filipino Nurses Association UK (FNAUK), social media, and email. Prospectively recruited individuals were provided with a participant information sheet and informed consent was recorded digitally using the web platform RedCap, prior to the interviews and focus groups being conducted.

### Data collection

We collected data through a brief demographic questionnaire, qualitative semi-structured interviews and focus groups. Due to the pandemic restrictions, interviews and focus groups were conducted online on Microsoft Teams by trained qualitative researchers (LBN, MG, AAO, FW and IQ). A piloted topic guide was used during the interviews and focus groups, which is provided in the Supplementary file. Interviews and focus groups lasted approximately 1.5 hours, with focus groups varying from 2 to 7 members. A gift voucher worth £20 was provided to the study participants in recognition of their time and contributions to the study. Interviews and focus groups were recorded with participants’ permission and were professionally transcribed and checked for accuracy by the research team.

### Analysis

We analyzed and coded transcript data using the thematic analysis (TA) approach of Braun and Clarke^19^ to identify key themes of lived experiences mentioned by the participants during their interviews. The authors (JC, IQ, LBN, MG and AAO) analyzed the data using an inductive approach to identify key themes emerging from the data, prompting the development of an iterative theoretical framework to improve understanding of how or if the COVID-19 pandemic affected and impacted reproductive and maternal health services. The transcripts were coded and analyzed collectively by team members using Microsoft Word to aid analysis between different partnership institutions. Following coding of the transcripts, emerging themes were discussed collaboratively between the team (JC, LBN, IQ, MG and AAO) and we finalized our interpretation of the emerging themes from the data in our regular weekly meetings until no new themes could be found from the data. The research team comprised a mixture of diverse backgrounds, including ethnicities (Black, South Asian, Middle Eastern and White), migrants, and both males and females. Their academic and professional backgrounds include, public health, health policy, education, anthropology, social work, medicine, and pharmacy. The process of bracketing^20^ was conducted to identify unconscious bias prior to data analysis and coding.

## RESULTS

### Demographic data

This study included a sample of 44 participants from the 164 UK-REACH participants. The study population included participants based on their job roles, as well as those who were either working directly with maternal and reproductive health service patients or reported encountering pregnant patients indirectly in their roles. Over three-quarters of recruited participants were from minoritized ethnic groups. Participants in the focus groups were identified by their pre-assigned code assigned during the focus group discussions.

Three key themes were identified from the data detailing the ways in which the UK Government’s COVID-19 policies and guidelines impacted MRH, staff and patients: 1) Infection control policies led to delays, cancellations of treatments, consultations and surgery. Appointments were also conducted online to reduce risks to HCWs; 2) Staff shortages, redeployment and additional workloads for senior clinicians, impacting consultations and surgery availability, reduced service quality and backlogs; and 3) HCWs and patients experienced challenges accessing trustworthy and timely COVID-19 vaccine information, increasing vaccine hesitancy among MRH HCWs and their patients. These themes are detailed in the following paragraphs.

**Table 1:** Participants’ demographic characteristics, United Kingdom, 2020–2021 (N=44)

*Characteristics*	*n*
**Sex**	
Male	12
Female	32
Age (years), median (range)	43 (20–66)
**Ethnicity**	
Asian*	15
Black**	8
Mixed	7
White	12
Other	2
**Job role**	
Doctors	13
Dentistry	3
Nurses	11
Midwives	3
Allied Health Professionals (AHP)	11
Administrative and other non-clinical	3

* Asian category includes all those under Asian/Asian British United Kingdom Census Categories (Indian/Pakistan/Bangladeshi/Chinese/Other Asian)^[Bibr cit0021]21^.

** Black category includes all those under Black/Black British United Kingdom Census Categories (African, Caribbean, any other Black, African, or Caribbean backgrounds)^21^.

### Impact of infection controls on patient services, consultations, and extended use of telemedicine

Infection control measures were implemented across all NHS Trusts to reduce the risks associated with face-to-face consultations. Non-urgent elective gynecological operations were cancelled or postponed. Virtual consultations were implemented through the use of telemedicine, which presented a disadvantage to some patients:

*‘So, I changed jobs from a combined obs/gynae job to a pure gynecology job starting the first week in April and so the Trust at that time had started to limit clinical activity, so we were stopped from face-to-face clinics to having virtual consultations. So, I would see about 90% of my patients virtually, so usually by telephone. Theatre lists were pretty much reduced. I would do two and a half theatre lists every week but that was reduced to maybe one a week or one every two weeks. So more patients having a disadvantage from that.’* (Doctor, WP4.FG04 M)

Where consultations could not be conducted virtually, a strict time limit of 15 minutes on face-to-face consultations was implemented:

*‘We weren’t allowed to have patients in with us in the clinic room for longer than 15 minutes, which meant that we would have to do a preceding telephone consultation and then bring them in for a very limited objective physical examination, but they were only able to spend 15 minutes in with us, then we would have 15 minutes to aerate the room and complete all of the infection control, kind of wiping down the couch, or the treatment table, wiping down the chairs and the waiting area that the patient would have been in, there was a lot more wiping down than maybe we were used to, but we definitely – of course it was all necessary.’* (Allied Health Professional, WP4.201)

With many patients unable to access face-to-face consultations in primary care, other community care settings including Genitourinary Medicine (GUM) and Sexual and Reproductive Health (SRH) clinics, reported a surge in demand from patients who were unable to attend their GP for their SRH requirements:

*‘We saw nobody, we spoke to everybody on the phone and people were getting very frustrated because they couldn’t get their contraception, their GP wouldn’t talk to them either… So that created a lot of frustration with the patients, which then rubbed off on the staff. We have found a better way of working. We are talking to everybody over the phone and bringing in people who we need to see…we try and time it and how urgent is it, how important is it for you for your physical and for your mental health to be seen? … So, over the year I’ve come to the conclusion that a large part of our job in sexual health is to make sure that we are catching up on the people that the GP won’t see, but who feel they need us.’* (Nurse, WP4.FG09.F2)

Some midwives and obstetricians spoke of how they believed their Trusts should continue to restrict visitors to two per patient following the pandemic to control future infections. However, these views were not uniformly shared across all participants. Three participants felt that despite their risk of infection to staff and patients, visitors were an integral element of the recovery and wellbeing of their patients:

*‘If people are in for day surgery they don’t need to have four different people in visiting them. I feel like from that side of things, I feel visiting should stay restricted. I think that people should have visitors, definitely, but I think they should be restricted back to one person, maybe two persons. I know for us on the maternity wards, we have quite a high turnover, so people come in, they give birth and then they go home, sometimes they have a two day stay, but in the grand scheme of things it is a two day stay, so wait until you’re home until your visitors come in. So I think that should stay.’* (Midwife, WP4.190)

### Staff shortages, redeployments, and additional workload

MRH staff were redeployed to other departments, such as respiratory, acute medicine and Accident and Emergency (A&E). Some MRH junior doctors, nurses and midwives were redeployed to work in these other departments. This resulted in understaffed departments and wards with senior doctors including consultants working longer hours and filling in for jobs usually performed by junior doctors and midwives including antenatal care clinics:

*‘We have quite a number of junior trainees who were going to be moved to a completely different environment in order to support the Covid activity. It does mean that many of us will then have to step down in our respective consultant posts to do some registrar work or, even worse, we might have to be doing our clinics and antenatal clinics all by ourselves with little or no support from the middle grade. And that also relates to staffing level as well – and the reason why the clinics were more or less transformed completely…to allow some of the supporting staff, like the healthcare assistants and nurses, to be moved to other departments that have got the bigger impact of Covid, particularly in respiratory medicine, Accident & Emergency and acute medical services.’* (Doctor, WP4.101)

A sonographer working in maternal health explained that her department was short-staffed, running at just 20% staff capacity, which impacted the quality of care received by patients:

*‘Our rota has changed significantly because of so many staff getting corona or having to isolate or schools shutting. A lot proportion of our staff are ladies with young children so I think normally we have, say 40-50 sonographers working and I think that first week in January when the second wave hit, I think there was only 10 sonographers in at one point, which is just madness really when you think about it.’* (Allied Health Professional, WP4.FG13 F2)

### Challenges of accessing information

Eleven participants emphasized the challenges of accessing reliable information about the COVID-19 vaccine’s safety and impact on the unborn fetus, fertility rates, and breastfeeding mothers. At times, information was confusing and contradictory. Some HCWs were as uninformed as the patients they were trying to help. These issues were further compounded due to the rapidly changing government guidance and lack of published research literature:

*‘No, so all the staff that I know have had it, and the only issue that we’re having at the moment now is that pregnant women are being advised to have it, so we’re getting a lot of questions there because obviously they’re worried about long term effects, which is understandable, the long-term effects of the vaccine. But like we say to them, we don’t know – yes, OK it’s not been trialed for five years or ten years and we don’t know the long-term effects but we know it’s safer than having Covid when you’re pregnant.’* (Midwife, WP4.190)

Guidance was often issued from various sources, multiple times throughout the day by various sources. Staff reported that these guidelines were confusing and sometimes contradictory. As a result, staff struggled to keep up with these rapidly changing updates and did not always know which guideline to follow:

*‘Infection control would tell us something different, management would tell us something different, so it was like, “What are we following?” And then it was, “No, that bulletin was from this morning, we’ve got a new one that came out at two o’clock this afternoon”. But then you’d read the latest one and it’s, “Well, no, that’s not what infection control said”…’* (Midwife, WP4.041)

## DISCUSSION

In this study, we captured the lived experiences of HCWs in maternal and reproductive health during the COVID-19 pandemic. We explored how the UK Government’s COVID-19 policies, restrictions and guidelines impacted maternal and reproductive healthcare services during the pandemic. The participants selected in this study were from a variety of specialities including obstetrics and gynecology, general practice, radiology, pharmacy, and dentistry. This diverse sample allowed us to understand how the pandemic affected pregnant women during their interactions with various healthcare specialities and departments during the pandemic. Our analysis provides valuable insights for researchers and policy makers, offering a comprehensive understanding of how staff and patients in maternal and reproductive healthcare were impacted by the policies and guidelines of the pandemic.

Three main themes emerged from the data: the impact of the pandemic on patient services due to infection controls; staff shortages, redeployments, and additional workloads; and challenges of accessing information and guidelines.

During the pandemic restrictions led to the cancellation of and delays to elective, non-life-threatening surgery^22^. In gynecological departments participants found that staff frequently outnumbered patients admitted to the ward. With fewer patients referred to gynecological departments and spare staff capacity, staff believed that their departments had the extra capacity to safely provide more elective gynecological treatments to patients, especially considering that staff were already working in these settings but had no patients to provide care for. These cancellations and delays may have contributed to longer term worsening health outcomes, risks to patient safety and contributed towards the surgery ‘backlog’^23^ which according to the British Medical Association reached a peak level of 7.19 million people in September 2022^24^ .

Our findings are consistent with previous literature reports that infection control policies included restricted visitor policies within maternity units across the NHS^16,25^. Although our participants acknowledged that visitors were an important element of the healing process, they did not explicitly comment on religious or cultural discrimination related to birthing practices requiring family members to be present during labor. Previous research has shown that accommodating specific religious and cultural birthing practices is linked with improved health outcomes^26^. Additionally, studies^15,16^ have highlighted that some minoritized ethnic patients opted paying out-of-pocket expenses for childbirth, so that their preferences were met. The significance of our results, supporting the existing literature that these policies were present, underscores the importance of these policies in relation to health inequalities arising from intersecting factors such as^16,17^ ethnicities, religious beliefs and cultures within the UK.

Our results also support existing literature around the consistent use of telemedicine throughout the pandemic in both primary and antenatal care^27^. However, not all patients or staff found telemedicine an acceptable alternative to face-to-face consultations. Specifically, HCWs from GUM and SRH clinics where face-to-face consultations continued throughout the pandemic, reported increased demand from new maternal and reproductive health patients seeking face-to-face appointments which were not offered by their GP. Other literature suggests that pregnant women from ethnic minority backgrounds felt that the shift to online antenatal classes was discriminatory and lacked sensitivity to their cultural and religious needs of privacy^28^.

### Strengths and limitations

This study included a national study sample of healthcare providers from various specialties, ethnicities and backgrounds with experience of working with maternal and reproductive health patients during the COVID-19 pandemic. By studying their lived experiences, we can holistically understand the ways in which the pandemic policies and guidelines affected maternal and reproductive healthcare.

The study was conducted retrospectively using an embedded analytical approach to examine the impacts of the pandemic’s policy and guideline implications on maternal and reproductive healthcare. While this approach has enabled us to examine the lived experiences of these healthcare workers during the pandemic to aid policy makers in future pandemic preparedness, it is important to acknowledge that interview responses may have lacked depth and themes identified may not be fully representative for the question explored. This limitation could be attributed to certain topics not arising during interviews or fulfilling the authors’ criteria for data saturation. Importantly, we acknowledge that the availability and flexibility of our participants during the pandemic for interviewing may have unintentionally led to selection and recruitment bias of study participants. We acknowledge that the selection of quotes may not reflect the full range of participant viewpoints. Therefore, an analysis of variation across different participants viewpoints including diversity of job role, ethnicity and sex maybe lacking. However, despite these limitations the study’s overall findings significantly contribute to our understanding of the challenges and impacts experienced by healthcare providers in maternal and reproductive health during the COVID-19 pandemic.

### Implications

The findings of this study provide a first-hand lived experience of the implications and impacts of the COVID-19 policies and guidelines on maternal and reproductive health patients and staff. This study highlights the need for a future respiratory pandemic preparedness policy and guidelines to include the following recommendations to improve access to maternal and reproductive health:

1. *Tailored infection control policies*: NHS trusts and integrated care units should review and consider the implications of infection control policies to safeguard against disproportionately impacting vulnerable and minoritized ethnicities requiring specific cultural and religious care needs by consulting with public, patient informing (PPI) and advocacy groups. This study especially highlights the need for policy makers to consider consultations with the public for maternal and reproductive health needs in primary care and obstetrics departments, where telemedicine is not widely acceptable by all populations.
2. *Addressing elective surgery backlogs early*: This study highlights the spare capacity within the healthcare system to address elective surgery backlog issues early. We recommend that in future pandemics, the government prioritizes strategies to reduce patient waiting times for elective surgery backlogs by ensuring that staff redeployment policies allow for more elective surgeries to take place with closer coordination with private healthcare providers to address the potential future surgical backlogs.
3. *Vaccine information and research dissemination*: The study findings highlight the importance for clearer and easily accessible vaccine information for maternal and reproductive healthcare workers and their patients. We recommend enhancing communications of research literature to different audiences. Improving staff awareness and trust through better communications may help to reduce issues of vaccine hesitancy among nursing and midwifery staff.

## CONCLUSIONS

Our study demonstrates that the COVID-19 policies and guidance affected maternal and reproductive health services and patients throughout the pandemic. The UK government’s policy implementation and COVID-19 response to reduce the risks to maternal and reproductive healthcare patients and staff included strict public health infection control regulations designed to ‘protect the NHS’ by reducing the overburdening demand placed on the healthcare system and to focus its resources on improving COVID-19 patient outcomes. Despite this, the health needs of maternal and reproductive patients continued despite the pandemic. As such, we have identified that the implementation of many of the pandemic’s policies and guidelines enforced during this period likely contributed towards worsening health outcomes and reduced access to maternal and reproductive health care.

Some services were cancelled entirely, and other services were sometimes conducted virtually or postponed, contributing towards backlogs after the pandemic, further straining the operational sustainable output of the NHS. We also found evidence to demonstrate that redeployment of staff was poorly managed. Workload was sometimes disproportionately distributed and increased for some senior clinicians, meanwhile some departments where elective surgeries took place, such as in gynecology, wards were overstaffed with no patients for staff to treat.

## Data Availability

The data supporting this research are available from the authors on reasonable request.
